# Prognostic value of interim and end-of-treatment FDG-PET in follicular lymphoma: a systematic review

**DOI:** 10.1007/s00277-015-2553-2

**Published:** 2015-11-18

**Authors:** Hugo J. A. Adams, Rutger A. J. Nievelstein, Thomas C. Kwee

**Affiliations:** Department of Radiology and Nuclear Medicine, University Medical Center Utrecht, Heidelberglaan 100, 3584 CX Utrecht, The Netherlands

**Keywords:** End-of-treatment, FDG-PET, Follicular lymphoma, Interim, Systematic review

## Abstract

This study aimed to systematically review the prognostic value of interim and end-of-treatment ^18^F-fluoro-2-deoxy-d-glucose positron emission tomography (FDG-PET) in follicular lymphoma during and after first-line therapy. The PubMed/MEDLINE database was searched for relevant original studies. Included studies were methodologically assessed, and their results were extracted and descriptively analyzed. Three studies on the prognostic value of interim FDG-PET and eight studies on the prognostic value of end-of-treatment FDG-PET were included. Overall, studies were of poor methodological quality. In addition, there was incomplete reporting of progression-free survival (PFS) and overall survival (OS) data by several studies, and none of the studies incorporated the Follicular Lymphoma International Prognostic Index (FLIPI) in the OS analyses. Two studies reported no significant difference in PFS between interim FDG-PET positive and negative patients, whereas one study reported a significant difference in PFS between the two groups. Two studies reported no significant difference in OS between interim FDG-PET positive and negative patients. Five studies reported end-of-treatment FDG-PET positive patients to have a significantly worse PFS than end-of-treatment FDG-PET negative patients, and one study reported a non-significant trend towards a worse PFS for end-of-treatment FDG-PET positive patients. Three studies reported end-of-treatment FDG-PET positive patients to have a significantly worse OS than end-of-treatment FDG-PET negative patients. In conclusion, the available evidence does not support the use of interim FDG-PET in follicular lymphoma. Although published studies suggest end-of-treatment FDG-PET to be predictive of PFS and OS, they suffer from numerous biases and failure to correct OS prediction for the FLIPI.

## Introduction

Follicular lymphoma is the second most frequently occurring non-Hodgkin lymphoma subtype [1]. In contrast to the 2007 Revised Response Criteria for Malignant Lymphoma [2] that considered computed tomography (CT) as the standard method for therapy response assessment in follicular lymphoma, the recently published Lugano Classification recommends using ^18^F-fluoro-2-deoxy-d-glucose positron emission tomography (FDG-PET) in follicular lymphoma [3, 4]. Moreover, the current guidelines also advocate the use of interim FDG-PET during treatment, although they do no support changing therapeutic strategy on the basis of interim FDG-PET findings outside clinical trials [3, 4]. Despite the recommendations in the new guidelines, there is still considerable debate on the utility of FDG-PET for therapy response assessment in follicular lymphoma and, not surprisingly, wide variability in the use of FDG-PET in these settings among different institutions. This debate on and the variability in the application of FDG-PET in follicular lymphoma reflect the need for evidence-based data. Although individual studies can provide more insight, there may be variability among individual studies with regard to internal validity (i.e., risk of bias) and external validity (i.e., generalizability of the study results). To overcome the limitations of individual studies, a systematic review is required. The purpose of this study was therefore to systematically review published data on the prognostic value of interim and end-of-treatment FDG-PET in follicular lymphoma.

## Materials and methods

### Search strategy

A systematic literature search was performed in the PubMed/MEDLINE database using the search terms that are displayed in Table 1. The search covered the period from start date to March 20, 2015. The reference lists of all studies that were finally included were screened for potentially suitable articles that were not detected by the initial search.

**Table 1:** Search strategy and results as on March 20, 2015

No.	Search string	PubMed/Medline
1.	Fluorodeoxyglucose OR 2-fluoro-2-deoxy-d-glucose OR FDG OR 18F-FDG OR positron emission tomography OR PET	86,557
2.	Follicular OR indolent	62,338
3.	No. 1 AND No. 2	651

### Study selection

Original studies that reported on the prognostic value of interim or end-of-treatment FDG-PET in indolent (grade 1-3A) follicular lymphoma during or after first-line therapy were included. Only studies written in English, German, Italian, French, Spanish, or Dutch were included. Studies without patient data (guidelines, reviews, meta-analyses, editorials, and letters), studies including less than ten patients with follicular lymphoma, duplicate studies, conference abstracts, studies that did not allow data extraction of patients with follicular lymphoma patients from patients with other lymphoma subtypes, studies that included patients with refractory disease, studies in which patients received second-line therapy, and studies that tailored the therapy on the basis of the interim or end-of-treatment FDG-PET result were excluded. Titles and abstracts of all studies that were obtained by the PubMed/MEDLINE search were scrutinized, and clearly ineligible studies were excluded at this stage. The full-text versions of the remaining articles were then evaluated, and a final decision was made as to whether or not a study met the criteria to be included in the systematic review.

### Study quality

The Quality In Prognosis Studies (QUIPS) tool [5], which was developed to assess the risk of bias in prognostic studies, was used for the methodological quality assessment. The QUIPS tool assesses the risk of bias in six different domains: study participation (“does the study population adequately represent the population of interest?”), study attrition (“do the study data available [i.e., patients not lost to follow-up] adequately represent the study sample?”), prognostic factor measurement (“is the prognostic factor measured in a similar way for all participants?”), outcome measurement (“is the outcome of interest measured in a similar way for all participants?”), study confounding (“have important potential confounding factors appropriately been accounted for?”), and statistical analysis and reporting (“is the statistical analysis appropriate, and are all primary outcomes reported”) [5]. Risk of bias was scored “low,” “moderate,” or “high for these six domains.

### Data analysis

Results of included studies were extracted and descriptively analyzed.

## Results

### Literature search

The literature search revealed a total of 651 articles (Table 1). After screening titles and abstracts, 41 potentially relevant articles remained and were retrieved and read in full-text format. Of these 41 articles, 16 were excluded because the prognostic value of interim or end-of-treatment FDG-PET status was not reported, 6 were excluded because of inclusion of less than 10 patients with follicular lymphoma, 4 were excluded because data of patients with follicular lymphoma could not be separated from patients with other lymphoma subtypes, 4 were excluded because of (partial) inclusion of patients with refractory disease, 1 was excluded because it only included patients with transformed lymphomas, 1 was excluded because it reported data that were also used in another (larger) study, and 1 was excluded because it was written in Japanese. Thus, eight studies remained. Of these eight studies, three reported data on the prognostic value of interim FDG-PET and eight studies reported data on the prognostic value of end-of-treatment FDG-PET. Characteristics and applied methodology of the eight included studies are displayed in Tables 2 and 3.

**Table 2:** Characteristics of included studies and patients

Study (year)	Country	Data acquisition	No. of patients^a^	Age in years (range)	Sex (M/F)	Stage (no.)	FLIPI score^d^	Treatment regimes (no.)
Lu et al. [6] (2014)	Australia	Retrospective	57	59^b^ (27–77)	26/31	I, 3 II, 7 III, 24 IV, 21	0, 2 1, 9 2, 19 3, 11 4, 8 5, 4 Unavailable, 4	6× R-CHOP, 57 Additional maintenance R, 45
Luminari et al. [7] (2014)	Italy	Retrospective	202	56^b^ (33–75)	98/104	II, 16 III/IV, 186	0–2, 131 3–5, 71	R-CVP, 66 R-CHOP, 62 R-FM, 74
Zinzani et al. [8] (2013)	Italy	Retrospective	142	63^a^ (25–83)	55/87	III, 65 IV, 77	NR	R-FM
Dupuis et al. [9] (2012)	Multinational	Prospective	119	57^b^ (28–76)	63/56	I/II, 8 III/IV, 110	0–1, 17 2, 50 3–5, 49	6× R-CHOP, 113 2× additional R, 106
Trotman et al. [10] (2011)	Multinational	Prospective	122	57^b^ (26–82)	67/55	I/II, 14 III/IV, 108	0–1, 28 2, 47 3–5, 47	6× R-CHOP + 2× R, 103 8× R-CVP, 19
Le Dortz et al. [11] (2010)	France	Retrospective	45	60^a^ (47–78)	22/23	I/II, 5 III, 15 IV, 25	0–1, 9 2, 12 3–5, 24	6× R-CHOP
Bishu et al. [12] (2007)	USA	Retrospective	16	NR	NR	II, 3 III, 5 IV, 8	NR	CHOP, R-CHOP, CNOP, CVP, R-monotherapy with/without additional RT
Zinzani et al. [13] (2007)	Italy	Retrospective	45	55^b^ (31–78)	25/20	III/IV, 40	2, 28 ≥ 3, 17	R-FM or R-CHOP

*CHOP* cyclophosphamide, doxorubicin, vincristine, and prednisone; *CNOP* cyclophosphamide, mitoxantrone, vincristine, and prednisone; *CVP* cyclophosphamide, vincristine, and prednisone; *FM* rituximab, fludarabine, and mitoxantrone; *ICE* ifosfamide, carboplatin, etoposide; *NR* not reported; *R* rituximab; *RT* radiation therapy

^a^Number of patients who were included in this study of whom baseline characteristics were available

^b^Median

^c^Mean

^d^FLIPI score [14]

**Table 3:** CT and FDG-PET acquisition/interpretation and patient follow-up methods

Study (year)	Histological grade	FDG-PET imaging system(s)	FDG dose	Time between FDG administration and scanning	Time between last therapy cycle and FDG-PET (range)	Interpreters	Baseline scan available (no.)	Follow-up examinations to determine end of PFS	Definition of end of PFS
Lu et al. [6] (2014)	1-3A	Stand-alone PET (*n* = 15) or hybrid PET/CT (*n* = 42)	5.14 MBq/kg	60 min	NR	Experienced PET physicians	Yes (55/57 cases)	NR	Relapse or aggressive transformation
Luminari et al. [7] (2014)	NR	Hybrid PET/CT	NR	NR	36 days^a^ (10–92)	Local nuclear medicine physician scan report	Yes (155/202 cases)	NR	Progression, relapse, or death from any cause
Zinzani et al. [8] (2013)	Grade 1-3A	NR	NR	NR	NR	Local nuclear medicine physician scan report	Yes	CT every 6 months for the first 2 years, annual imaging studies with CT or FDG-PET/CT up to 7 years	Progression or death from any cause
Dupuis et al. [9] (2012)	1-3A	NR	3.5–8 MBq/kg	60 min	NR	Central review by 3 experienced nuclear medicine physicians	Yes (all)	Clinical examination every 3 months and CT scan every 6 months during the first 2 years	First documented evidence of progressive disease, relapse, or death resulting from any cause
Trotman et al. [10] (2011)	NR	Hybrid PET/CT	NR	NR	64 days^a^ (9–124)	Local nuclear medicine physician scan report	NR	Clinical examination every 3 months and CT scans every 6 months for 3 years	Progression/relapse (on the basis of the investigator's assessment) or death from any cause
Le Dortz et al. [11] (2010)	1-3a	Hybrid PET/CT	5 MBq/kg	60 min	NR	Two nuclear medicine specialists	Yes (all)	NR	Progression, relapse or second-line therapy
Bishu et al. [12] (2007)	1 or 2	Stand-alone PET or hybrid PET/CT	370–740 MBq	60 min	NR	Experienced nuclear radiologists	Yes (all)	Conventional methods every 3–6 months for 1 year, every 6–12 months for 2 years, FDG-PET examination every 3–12 months	Histologic, clinical or radiographic evidence of disease progression
Zinzani et al. [13] (2007)	1 or 2	Stand-alone PET	370 MBq	70–90 min	Approximately 30 days	Three experienced readers	Yes	CT and FDG-PET were performed every 6 months for the first 2 years and then every 12 months	Progression or relapse

*NR* not reported, *PFS* progression-free survival

^a^Median

### Methodological quality assessment

Results of the methodological quality assessment using the QUIPS tool are shown in Table 4. There was high risk of bias in one study for both the domains study participation and study confounding because this study only included cases that were clearly positive or negative and excluded cases that were not unequivocally positive or negative [7]. There was high risk of bias for the domain prognostic factor measurement in four studies [7, 8, 10, 13] because these studies did not report clear criteria for FDG-PET positivity/negativity, and there was moderate risk of bias for the domain prognostic factor measurement in three other studies because one study [9] did not report which PET system (modern hybrid PET/CT or diagnostically inferior stand-alone PET) was used for FDG imaging and two studies [6, 12] reported that (some of) the included patients underwent stand-alone FDG-PET. Finally, there was high risk of bias for outcome measurement in all eight studies [6–13], because none of the eight studies reported clear and reproducible criteria for disease progression/relapse/end of progression-free survival (PFS), three studies [6, 7, 11] did not report whether follow-up visits/examinations were standardized or similar in both the FDG-PET negative and positive group, and five of eight studies [7, 8, 11–13] failed to provide data on the overall survival (OS).

**Table 4:** Quality assessment of included studies according to the QUIPS tool [5]

Study (year)	Study participation	Study attrition	Prognostic factor measurement	Outcome measure	Study confounding	Statistical analysis
Lu et al. [6] (2014)	Low	Low	Moderate	High	Low	Low
Luminari et al. [7] (2014)	High	Low	High	High	High	Low
Zinzani et al. [8] (2013)	Low	Low	High	High	Low	Low
Dupuis et al. [9] (2012)	Low	Low	Moderate	High	Low	Low
Trotman et al. [10] (2011)	Low	Low	High	High	Low	Low
Le Dortz et al. [11] (2010)	Low	Low	Low	High	Low	Low
Bishu et al. [12] (2007)	Low	Low	Moderate	High	Low	Low
Zinzani et al. [13] (2007)	Low	Low	High	High	Low	Low

*Low* low risk of bias, *Moderate* moderate risk of bias, *High* high risk of bias

### Interim FDG-PET

The results of the three studies reporting on the prognostic value of interim FDG-PET are shown in Table 5. The proportion of interim FDG-PET negative and positive patients ranged from 64 to 76 % and 24.3 to 36.4 %, respectively. All three studies investigated the association of the interim FDG-PET result with PFS, with two studies reporting no significant difference in PFS between FDG-PET positive and negative patients [6, 12], whereas one study [9] reported FDG-PET positive patients to have a significantly worse PFS than FDG-PET negative patients [9]. Only two of three studies investigated the association of the interim FDG-PET result with OS, with both studies reporting no significant difference in OS between FDG-PET positive and negative patients [6, 9]. Of interest, none of the studies corrected the interim FDG-PET result for the predictive value of the Follicular Lymphoma International Prognostic Index (FLIPI) in the OS analyses.

**Table 5:** Results of studies reporting on the prognostic value of interim FDG-PET

Study (year)	Criteria for positivity	Follow-up time in months (range)	Number of therapy cycles before interim FDG-PET	No. of FDG-PET negative/positive patients	Number of relapses/progressions/events	PFS	Median/mean PFS (months)	Hazard ratio PFS	Deaths	OS	Median/mean OS (months)	Hazard ratio OS
Lu et al. [6] (2014)	Cheson/Barrington 2014 [3]/Deauville 4 + 5	42.2^a^ (7.8–103.8)	Generally 3 cycles	Negative, 28/44 (63.6 %) Positive, 16/44 (36.4 %)	NR	NR	Negative, 78.5 Positive, 51.0 (*P* = 0.35)	1.7 (95 % CI 0.5–5.5, *P* = 0.35)	NR	NR	Negative, 89.9 Positive, 76.6 (*P* = 0.92)	1.1 (95 % CI, 0.2–4.9, *P* = 0.92)
Dupuis et al. [9] (2012)	Cheson/Barrington 2014 [3]/Deauville 4 + 5	23^a^	4 cycles	Negative, 84/111 (75.7 %) Positive, 27/111 (24.3 %)	NR	2-year PFS: Negative, 86 % Positive, 61 % (*P* = 0.0046)	NR	NR	NR	No significant difference	NR	NR
Bishu et al. [12] (2007)	Cheson/Juweid 2007 [2]/>MBP	26^b^ (6–58)	NR	Negative, 7/11 (63.6 %) Positive, 4/11 (36.4 %)	NR	No significant difference	Negative, 30 Positive, 17^b^	NR	NR	NR	NR	NR

*CI* confidence interval, *MBP* mediastinal blood pool, *NR* not reported, *PFS* progression-free survival, *OS* overall survival

^a^Median

^b^Mean

### End-of-treatment FDG-PET

The results of the eight studies reporting on the prognostic value of end-of-treatment FDG-PET are displayed in Table 6. The proportion of end-of-treatment FDG-PET negative and positive patients ranged from 71.1 to 87.5 % and 12.5 to 28.9 %, respectively. Of the eight studies, six investigated the association of the end-of-treatment FDG-PET result with PFS, with five studies reporting FDG-PET positive patients to have a significantly worse PFS than FDG-PET negative patients [7–11] and one study reporting a non-significant trend towards a worse PFS for FDG-PET positive patients [6]. Yet, another two studies did not report whether there was any significant difference in PFS between FDG-PET positive and negative patients [12, 13]. Only four of eight studies investigated the association of the end-of-treatment FDG-PET result with OS, with three studies reporting FDG-PET positive patients to have a significantly worse OS than FDG-PET negative patients [6, 9, 10], whereas one study did not report whether there was any significant difference in OS between FDG-PET positive and negative patients [7]. Of interest, none of the studies corrected the end-of-treatment FDG-PET result for the predictive value of the FLIPI in the OS analyses.

**Table 6:** Results of studies reporting on the prognostic value of end-of-treatment FDG-PET

Study (year)	Criteria for positivity	Follow-up time in months (range)	No. of FDG-PET negative/positive patients	Number of relapses/progressions/events	PFS	Median/mean PFS (months)	Hazard ratio PFS	Deaths	OS	Median/mean OS (months)	Hazard ratio OS
Lu et al. (2014) [6]	Cheson/Barrington 2014 [3]/Deauville 4 + 5	42.2^a^ (7.8–103.8)	Negative, 39 (83.0 %) Positive, 8 (17.0 %)	NR	3-year PFS Negative, 72 % Positive, 30 %	Negative, 74.4 Positive, 38.2 (*P* = 0.083)^b^	2.6 (95 % CI, 0.7–9.1) (*P* = 0.083)	NR	3-year OS: Negative, 96 % Positive, 60 %	Negative, 95.2 Positive, 45.0 (*P* < 0.001)	10.8 (95 % CI, 2.8–41.6, *P* < 0.001)
Luminari et al. (2014) [7]	NR	34^a^ (7–66)	Negative, 153 (75.7 %) Positive, 49 (24.3 %)	NR	3-year PFS: Negative, 66 % Positive, 35 % (*P* < 0.001)	NR	PFS, 2.59, 95 % CI, 1.59–4.24, *P* < 0.001)	Negative, 3/153 (2.0 %) Positive, 3/49 (6.1 %)	NR	NR	NR
Zinzani et al. (2013) [8]	NR	48^a^ (6–145)	NR	NR	5-year PFS: Negative, 75.5 % Positive, 42 % (*P* = 0.002)	NR	NR	NR	NR	NR	NR
Dupuis et al. [9] (2012)	Cheson/Barrington 2014 [3]/Deauville 4 + 5	23^b^	Negative 83/106 (78.3 %) Positive, 23/106 (21.7 %)	NR	2-year PFS: Negative, 87 % Positive, 51 % (*P* < 0.001)	NR	NR	NR	2-year OS, Negative, 100 % Positive, 88 % (*P* = 0.0128)	NR	NR
Trotman et al. 2011 [10]	NR	42^b^ (6–57)	Negative, 90 (73.8 %) Positive, 32 (26.2 %)	NR	42-month PFS: Negative, 70.7 % Positive, 32.9 % (*P* < 0.001)	Negative, not reached Positive, 20.5^a^	3.3 (95 % CI, 1.9–5.9)	Negative, 3/90 (3.3 %) Positive, 7/32 (21.8 %)	42-month OS: Negative, 96.5 % Positive, 78.5 % (*P* = 0.0011)	NR	7.0 (95 % CI, 1.8–27.0, *P* = 0.0011)
Le Dortz et al. [11] (2010)	Cheson/Juweid 2007 [2]/>MBP	36^a^ (24–50)	Negative, 32/45 (71.1 %) Positive, 13/45 (28.9 %)	Negative, 5/32 (15.6 %) Positive, 11/13 (84.6 %) (5× second-line treatment initiation, 6× progression)	NR	Negative, 48 Positive, 17.2 (*P* < 0.0001)	NR	NR	NR	NR	NR
Bishu et al. [12] (2007)	Cheson/Juweid 2007 [2]/>MBP	28^b^ (6–59)	Negative, 14/16 (87.5 %) Positive, 2/16 (12.5 %)	Negative, 1/14 (7 %) Positive, 2/2 (100 %)	NR	Negative, 29.5 months Positive, 5.8^b^	NR	NR	NR	NR	NR
Zinzani et al. [13] (2007)	NR	25^a^ (20–34)	Negative, 34/45 (75.6 %) Positive, 11/45 (24.4 %)	Negative, 2/34 (5.9 %) Positive, 10/11 (90.9 %)	NR	NR	NR	NR	NR	NR	NR

*CI* confidence interval, *MBP* mediastinal blood pool, *NR* not reported, *PFS* progression-free survival, *OS* overall survival

^a^Median

^b^Mean

## Discussion

This systematic review included three studies on the prognostic value of interim-FDG-PET and eight studies on the prognostic value of end-of-treatment FDG-PET in follicular lymphoma. The results show that there is a lack of data supporting the use of interim FDG-PET for the evaluation of first-line therapy in follicular lymphoma, given the fact that interim FDG-PET was reported to be not predictive of PFS in two of three studies and was not found to be predictive of OS in the two available studies on this topic. On the other hand, end-of-treatment FDG-PET seems to have some predictive value based on the available studies, with five studies reporting a significant and one study reporting a nearly significant association with PFS and three studies reporting a significant relationship between end-of-treatment FDG-PET status and OS.

Importantly, however, these results should be interpreted very cautiously due to the overall poor methodological quality of included studies. There was high risk of bias with regard to study participation and confounding in one study (due to the exclusion of unequivocal FDG-PET cases), moderate and high risk of bias with regard to prognostic factor measurement in three and four studies, respectively (due to the [possible] use of diagnostically inferior stand-alone PET systems and lack of reporting of FDG-PET interpretation criteria, respectively), and high risk of bias with regard to outcome measurement in all eight studies. The latter is due to several important reasons, the first being the fact that the reference standard may have been different between FDG-PET positive and negative patients in three studies. This is of particular concern in retrospective studies, because it is not uncommon that patients with residual disease according to FDG-PET are monitored more closely, with more follow-up visits and more follow-up imaging and laboratory examinations, which will result in an earlier detection of (asymptomatic) progressions in this group compared to the FDG-PET negative group. This may have seriously biased the reported prognostic values of FDG-PET. Second, none of the included studies reported clear and reproducible criteria for disease progression/relapse/end of PFS. Various definitions are used for end of PFS in follicular lymphoma: first clinical/radiological evidence of disease after attaining complete remission (not applicable in end-of-treatment FDG-PET positive cases), increase in FDG avidity at follow-up FDG-PET, increase in tumor volume at follow-up CT, alterations in laboratory assessments, development of symptomatic disease, histological evidence of high-grade disease transformation, and initiation of second-line therapy or death. It should also be noted that the majority of included studies only reported the PFS and not the OS or the interval between end-of-treatment FDG-PET and initiation of the next therapy cycle. End of PFS, when defined as (asymptomatic) disease progression detected by radiological studies, is not a sufficient indication for second-line treatment initiation or changing patient management otherwise. None of the studies reported data on the value of FDG-PET in predicting the interval between end-of-treatment and (re)development of symptomatic disease or time interval between the end-of-treatment and the initiation of a subsequent second-line therapy, which can be regarded as perhaps more important outcome measures. Thus, it should be emphasized that the definition of PFS that is employed by many studies is not the most important outcome measure in this disease entity, and that although the results of this systematic review may suggest that a positive end-of-treatment FDG-PET may be associated with a worse PFS, it does not support the use of end-of-treatment FDG-PET in routine clinical practice. Overall, the subjectivity of the outcome measure PFS and the fact that the PFS do not provide very clinically relevant data underline that future studies should focus in particular on the OS or the time interval to develop subsequent symptomatic disease requiring second-line therapy. Finally, and surprisingly, none of the included studies corrected the end-of-treatment FDG-PET result for the predictive value of the FLIPI [14] in the OS analyses. Until end-of-treatment FDG-PET is proven to be predictive of OS, independently of the (less expensive) FLIPI, it cannot be recommended yet for routine use in clinical practice.

There are several factors that make the role of FDG-PET in follicular lymphoma more devious in contrast to that in aggressive non-Hodgkin lymphomas and Hodgkin lymphoma. First, follicular lymphoma is an incurable disease as a result of which absence of viable tumor at FDG-PET never signifies absence of residual tumor cells but rather that the majority of the tumor bulk has responded and made the lymphoma undetectable by FDG-PET. Disappearance of lymphoma from the “FDG-PET radar”, may indicate a long-term (asymptomatic) remission, but not cure. Second, since viable tumor at FDG-PET in follicular lymphoma is not a direct indication for treatment initiation [1], detection of residual disease at end-of-treatment FDG-PET may be less relevant in follicular lymphoma than in aggressive non-Hodgkin lymphoma and Hodgkin lymphoma where residual disease after first-line therapy will often result in rapid additional diagnostic work-up with eventual consolidation radiation therapy or initiation of second-line therapies [3, 4, 15]. Third, in follicular lymphoma, bone marrow involvement is present in approximately 50 % of patients [14]. However, FDG-PET has proven to be inadequately sensitive for the detection of bone marrow involvement in follicular lymphoma [11, 16]. This in turn proves that FDG-PET cannot be used either for the evaluation of response to treatment of follicular lymphoma that lodges in the bone marrow. Fourth, in contrast to the more aggressive histologies, determination of the prognostic value of interim and end-of-treatment FDG-PET for predicting OS requires very long follow-up times, and results are not rarely biased by variations in the initiation, nature and intensity of post-first-line therapy, and other, non-lymphoma-related causes of death.

The present systematic review had limitations. First, it was not possible to meta-analyze the results of individual studies, because there was considerable variation in data reporting, and hazard ratios for PFS and OS were not reported in the majority of included studies. Second, only a minority of studies reported the value of FDG-PET in predicting OS and no study reported data on the time to subsequent treatment initiation of second-line therapy or time interval to (re)develop asymptomatic disease after treatment. Third, applied treatment regimens among the included studies were heterogeneous, which affects interstudy comparisons. Fourth, the included studies reported follow-up times ranging between 6 and 104 months, which is insufficiently long considering the generally prolonged survival of patients with follicular lymphoma after first-line treatment.

In conclusion, the available evidence does not support the use of interim FDG-PET in follicular lymphoma. Although published studies suggest end-of-treatment FDG-PET to be predictive of PFS and OS, they suffer from numerous biases and failure to correct OS prediction for the FLIPI.
